# Serum magnesium levels and lung cancer risk: a meta-analysis

**DOI:** 10.1186/s12957-018-1447-x

**Published:** 2018-07-12

**Authors:** Xinghui Song, Xiaoning Zhong, Kaijiang Tang, Gang Wu, Yin Jiang

**Affiliations:** 1 Department of respiration, the First Affiliated Hospital of Guangxi Medical University, N0.6 Shuangyong Road, Nanning, 530021 Guangxi China; 2 Department of rheumatism, Liuzhou Worker’s Hospital, Liuzhou, 545005 Guangxi China; 3 Department of neurosurgery, Liuzhou General Hospital, Liuzhou, 545006 Guangxi China

**Keywords:** Magnesium level, Lung cancer, Meta-analysis, Healthy controls

## Abstract

**Background:**

Whether serum magnesium levels were lower in patients with lung cancer than that in healthy controls is controversial. The aim of this study was to identify and synthesize all citations evaluating the relationship between serum magnesium levels and lung cancer.

**Methods:**

We searched PubMed, WanFang, China National Knowledge Internet (CNKI), and SinoMed databases for relevant studies before December 31, 2017. Two authors independently selected studies, extracted data, and assessed risk of bias.

**Results:**

Eleven citations comprising 707 cases with lung cancer and 7595 healthy controls were included in our study. Serum magnesium levels were not significantly lower in patients with lung cancer [summary SMD = 0.193, 95%CI = − 1.504 to 1.890] when compared to health controls, with significant heterogeneity (*I*^2^ = 99.6%, *P* < 0.001) found. Negative associations were found among Asian populations [summary SMD = 0.229, 95%CI = − 1.637 to 2.094] and European populations [summary SMD = − 0.168, 95%CI = − 0.482 to 0.147]. No publication bias was found using the test of Egger and funnel plot.

**Conclusions:**

Our study suggested that serum magnesium levels had no significant association on lung cancer risk.

## Background

Lung cancer is the leading cause of death from cancer, resulting 1.38 million people deaths each year [1]. Its 5-year survival rate is still as low as 15%, and it is poor while compared with those in high incidence of other cancer [2]. Previous studies pointed out that lung cancer is the most common cancer among men and women, and both developed and developing countries bear a huge social and economic burden [3]. Previous publications proved that both genetic and environment factors were related to lung cancer risk [4–7]. Furthermore, trace-heavy elements also played a significant role on human health and disease [8, 9], as well as lung cancer [10].

Magnesium is one of the trace elements in our bodies, and to date, some papers had been published to investigate the association between serum magnesium levels and lung cancer risks. Two papers [11, 12] reported a higher of serum magnesium level in cases with lung cancer, while six papers [13–18] found a lack of significant association. Conversely, three papers [19–21] suggested that it is lower in lung cancer cases when compared to the healthy controls. Therefore, the aim of this study was to identify and synthesize all citations evaluating the relationship between serum magnesium levels and lung cancer risk.

## Methods

### Study selection

A comprehensive literature search was conducted in platforms of PubMed, WanFang, China National Knowledge Internet (CNKI), and SinoMed databases up to December 31, 2017. Free words adopted were as follows: “magnesium” or “Mg” combined with “lung cancer” or “lung carcinoma” without restrictions. Reference lists of the studies retrieved were also examined to find any additional study potentially unidentified. The course of study selection was completed by two investigators independently. Any resulting discrepancies were resolved by a third reviewer.

The inclusion criteria were as follows: (i) having a prospective design or a case-control design or a cross-sectional study; (ii) evaluating the association between serum magnesium levels and risk of lung cancer; (iii) reporting mean and standard deviation (SD) of magnesium levels (or sufficient data to compute them) both in lung cancer patients and healthy controls; and (iv) studies published in English language or Chinese language. If more than one article referred to the same populations, only the study that included the most lung cancer cases or the latest publication was included.

### Data extraction and quality assessment of studies

Two investigators independently extracted the following data: (1) first author’s last name; (2) publication year; (3) study design; (4) country; (5) number of lung cancer cases and participants; (6) sex of cases; (7) age range or mean age of the cases; (8) mean and SD of magnesium levels both in lung cancer patients and healthy controls; and (9) method used for detection of magnesium. Any resulting discrepancies were resolved by a third reviewer.

The methodological quality of studies was evaluated independently by two researchers using the Newcastle-Ottawa Quality Assessment Scale [22]. The three components were as follows: (1) patient selection (4 points); (2) comparability (2 points); and (3) outcome (3 points) for a total score of 9 points.

### Statistical analysis

Standardized mean difference (SMD) and their 95% confidence interval (CI) were calculated for relationship between serum magnesium levels and lung cancer risk. A random effect model was used in our meta-analysis [23]. The heterogeneity among studies was evaluated with *I*^2^ and Q tests. [24]. *P* < 0.05 in Q test and *I*^2^ > 50% indicated statistically significant heterogeneity [25]. Meta-regression was adopted to assess the between-study heterogeneity. Egger’s regression asymmetry test [26] and funnel plot [27] were used to visually examine publication bias on study outcome. Statistical analyses were performed using STATA version 12.0 (Stata Corporation, College Station, TX, USA). A two-sided *P* < 0.05 was defined as statistical significance.

## Results

### Study characteristics

**Fig. 1:**
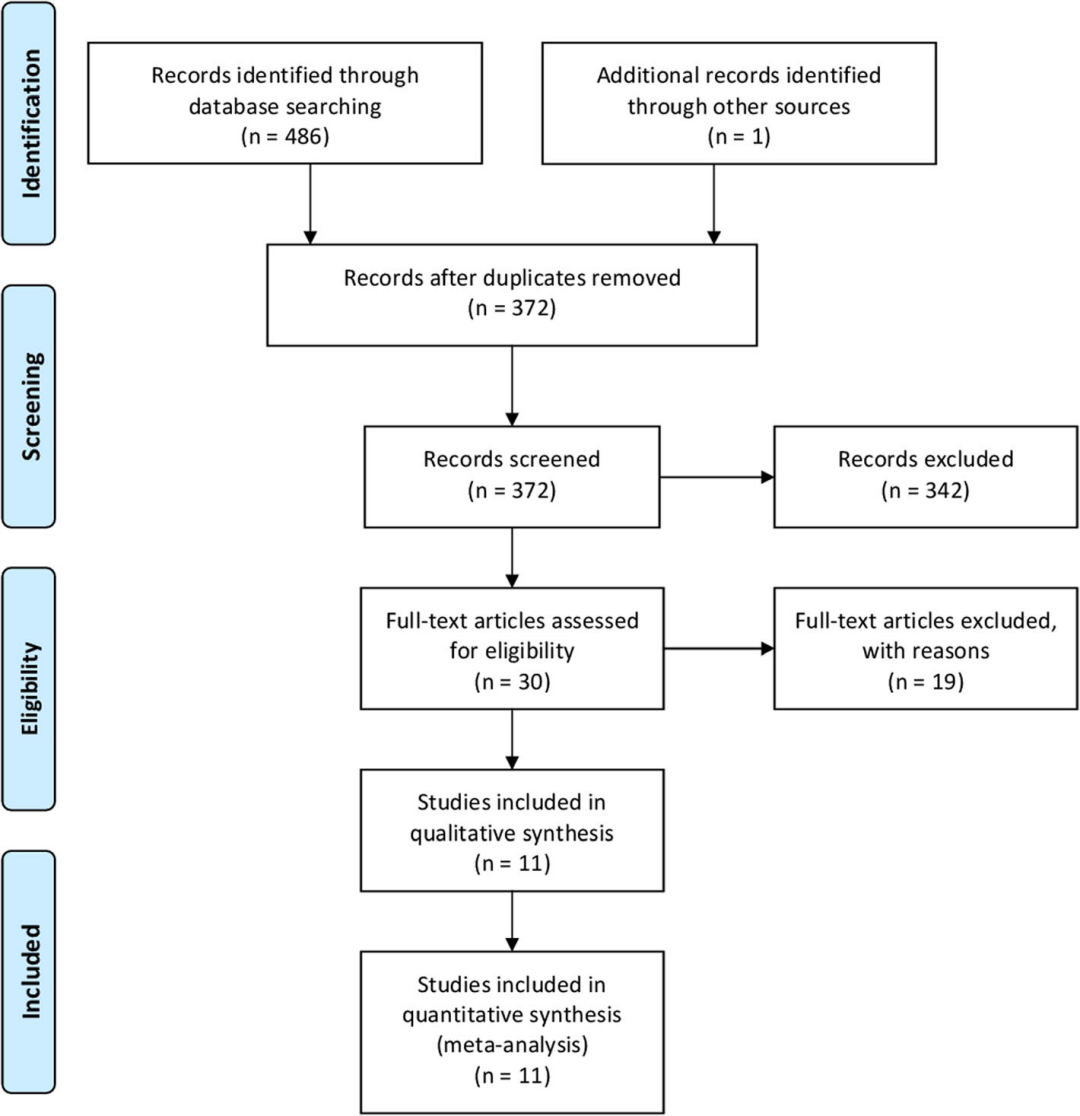
Flowchart of meta-analysis for exclusion/inclusion of studies

As shown in Fig. 1, the initial 486 articles screened through databases of PubMed, WanFang, China National Knowledge Internet (CNKI), and SinoMed databases searching and 1 additional record identified through other sources. There are 372 articles that were reviewed in the title and abstract while excluding the duplications from different databases. Three hundred and forty two of 372 articles were excepted when screened on the basis of title and abstract; 30 articles were examining full texts. Nineteen studies were further excluded (reviews, not report mean or SD, animal studies, letter to the editors). Finally, 11 articles [11–21] were eligible to be included in the analysis comprising 707 patients with lung cancer and 7595 healthy controls. All the included studies were case-control studies. Nine studies were carried out from China, 1 from Spain, and 1 from Turkey. Ten of the included studies used the methods of atomic absorption spectrophotometer measurements for detection of magnesium. In the study quality assessment, all the included studies were with a score greater or equal to 6. The basic features of all citations are shown in Table 1.

**Table 1:** Characteristics of all included studies

Study, year	Country	Age (range or Mean ± SD)	Study type	Lung cancer cases			Controls		Methods of measured magnesium
				*n*	Female (%)	Magnesium: Mean ± SD	*n*	Magnesium: Mean ± SD	
Cobanoglu U et al., 2010	Turkey	54 ± 8.29	Case-control	30	33.33	156.21 ± 22.21 μg/L	20	185.8 ± 4.05 μg/L	Atomic Absorption Spectrophotometer measurements (UNICAM-929 spectrophotometer)
Diez M et al., 1989	Spain	60 ± 7	Case-control	64	7.81	20.6 ± 3.2 μg/L	100	21.7 ± 8 μg/L	Perkin-Elmer 5.000 atomic absorption spectrophotometer
Jin ZJ et al., 2001	China	45–70	Case-control	40	7.50	1300 ± 390 μmol/L	46	1320 ± 310 μmol/L	Atomic Absorption Spectrophotometer measurements
Xu ZF et al., 1993	China	56 ± 7.5	Case-control	42	9.52	804.63 ± 71.29 μmol/L	40	936.83 ± 93.31 μmol/L	Atomic Absorption Spectrophotometer measurements
He WD et al., 1995	China	34–72	Case-control	143	39.16	940.88 ± 116.95 μmol/L	50	871.24 ± 96.88 μmol/L	Atomic Absorption Spectrophotometer measurements
Huang ZY et al., 1998	China	25–65	Case-control	136	19.12	1.8275 ± 0.375 μmol/L	7101	0.8254 ± 0.1778 μmol/L	Atomic Absorption Spectrophotometer measurements (Japan Shimadzu-AA670/C2H2)
Wang ZL et al., 2003	China	28–69	Case-control	50	40.00	68.29 ± 35.26 μg/L	60	114.1 ± 52.12 μg/L	Atomic Absorption Spectrophotometer measurements and 721 spectrophotometer
Du FL et al., 1996	China	22–73	Case-control	73	31.51	1100 ± 300 μmol/L	63	1100 ± 100 μmol/L	Atomic Absorption Spectrophotometer measurements
Guo XH et al., 1994	China	55.1	Case-control	26	26.92	20.88 ± 6.72 μg/mL	26	18.84 ± 5.86 μg/mL	Atomic Absorption Spectrophotometer measurements (Varian Spectr AA-40p, USA)
Wang FJ et al., 2014	China	17–77	Case-control	68	44.12	880 ± 60 μmol/L	60	860 ± 90 μmol/L	Xylene blue method
Fang JQ et al., 1998	China	55–65	Case-control	35	5.71	1.34 ± 0.35 μmol/L	29	1.36 ± 0.29 μmol/L	Atomic Absorption Spectrophotometer measurements

### Serum magnesium levels and lung cancer risk

**Fig. 2:**
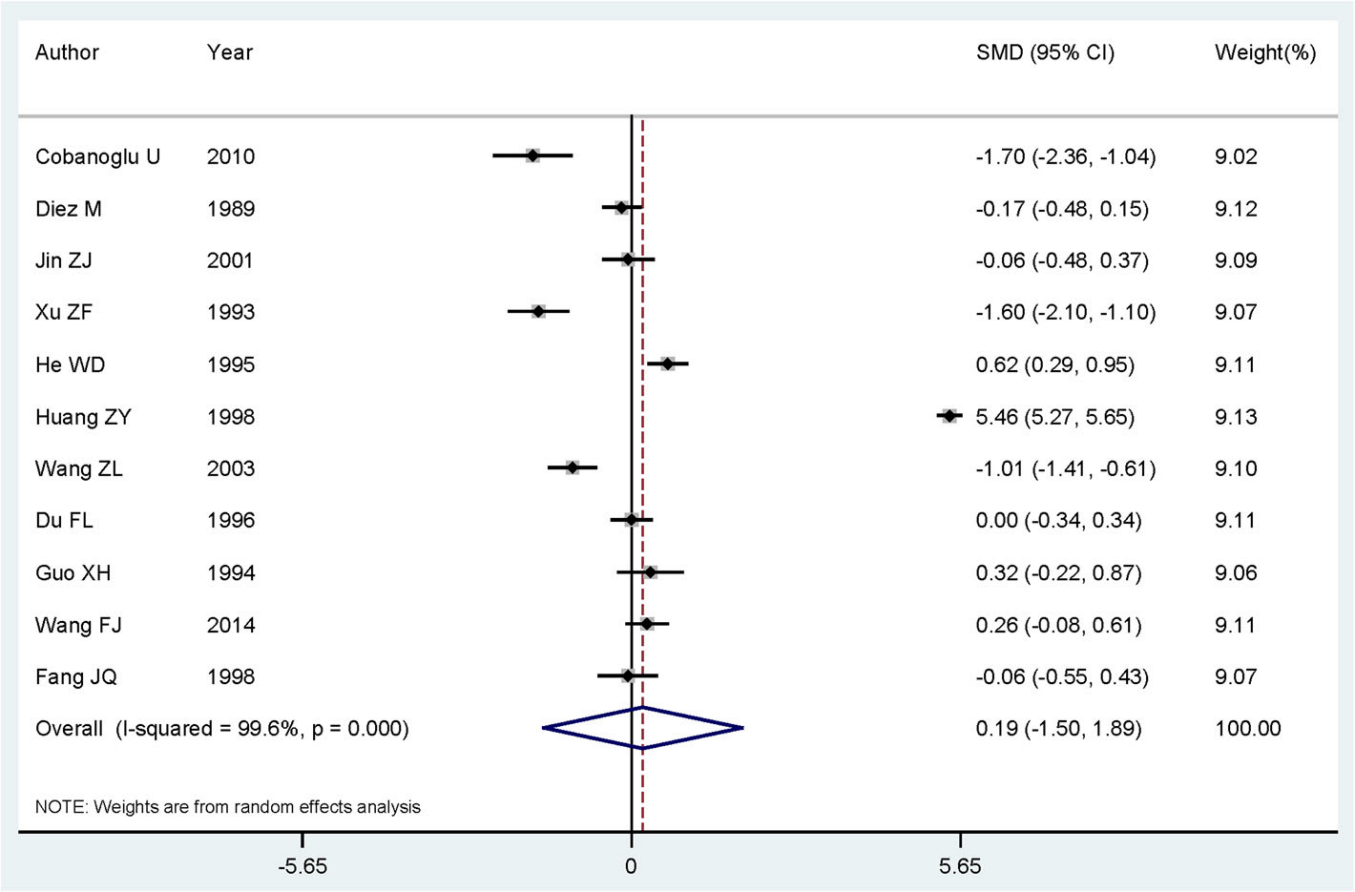
The forest plot of the relationship between serum magnesium levels and lung cancer risk

Pooled results suggested that magnesium levels in patients with lung cancer was not significantly lower than healthy controls [summary SMD = 0.193, 95%CI = − 1.504 to 1.890, *I*^2^ = 99.6%, *P*_for heterogeneity_ < 0.001] (Fig. 2). When we performed the subgroup analysis by geographic location, the association was not significant either in Asian populations [summary SMD = 0.229, 95%CI = − 1.637 to 2.094] or in European populations [summary SMD = − 0.168, 95%CI = − 0.482 to 0.147].

### Sources of heterogeneity and meta-regression

Meanwhile, *I*^2^ was 99.6% (*p* < 0.001) for the pooled sensitivity, suggesting high heterogeneity in the sample of studies. Univariate meta-regression was then carried out to determine the reason of heterogeneity. However, there were no significant contributions about publication year, case number, geographic location, sex, and different methods on this high between-study heterogeneity.

### Sensitivity analysis and publication bias

**Fig. 3:**
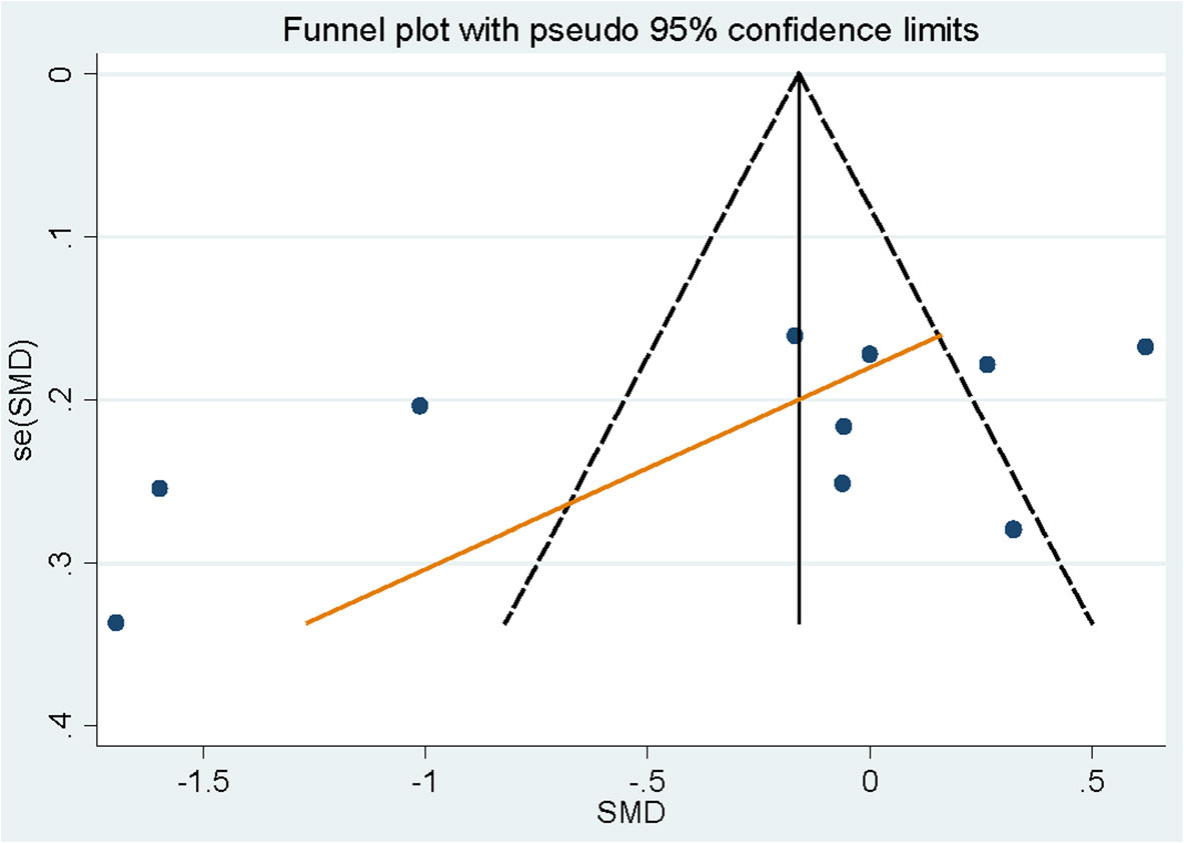
Funnel plot for the analysis of publication bias between serum magnesium levels and lung cancer risk

Sensitivity analysis conducted while removing one study at the time revealed that no single study had essential effect on the whole result. Figure 3 showed that no publication was considered by the funnel plot method on the basis of data, as well as the Egger’s test (*P* = 0.586).

## Discussion

In this study, we assessed the association between serum magnesium levels and risk of lung cancer. We did not find a positive association between serum magnesium levels and lung cancer risk. Through our subgroup analysis, we further found no significant association among Asian and European populations. Significant heterogeneity between studies observed in this meta-analysis should be considered as a major limitation of these findings; however, heterogeneity was mainly related to strength of the association rather than the direction of risk estimate, suggesting overall promising findings on the outcome investigated in the present study.

Two previous prospective cohort studies concluded that higher category of dietary magnesium intake had no significant association on lung cancer risk among German population and China population [28, 29]. However, a report [30] had been resulting that higher magnesium levels in drinking water could reduce the risk of lung cancer deaths in women. To our knowledge, no comprehensive analysis had been published to assess the serum magnesium levels on lung cancer risk. In our study, we did not find significant association of lower serum magnesium levels in patient with lung cancer. However, level of magnesium in other disease may be in the normal range, and that magnesium can have an effect on this disease [31].

The existence of heterogeneity among the studies, which is common in meta-analyses [32], may affect the pooled results. Meta-regression was performed to find the potential covariates (publication year, case number, geographic location, sex, and different methods to detect magnesium levels) which may cause this high heterogeneity. However, no covariate was found to significantly contribute to heterogeneity. In our study, most of the included studies obtained nonsignificant association between serum magnesium levels and lung cancer risk. Only one study [12] reported that serum magnesium level in patient with lung cancer is extremely higher than that in healthy controls. We reviewed the article again and confirmed the data exacted from the study; no error was made. Sensitivity analysis was performed, and no study had essential effect to the significant between-study heterogeneity and the whole result. On the other hand, we used a random effect model to combine the results. As we all know, random effect model had wider rage about 95%CI than fix effect model and could obtain more accurate results. Furthermore, only three studies [12, 13, 17] reported the types and staging of lung cancer, which may also be a factor on the between-study heterogeneity. Therefore, studies with detailed information of types and staging of lung cancer are wanted to further explore this association.

Some advantages existed in our study. Firstly, a comprehensive literature search was performed to investigate the relationship between serum magnesium levels and lung cancer risk. Secondly, most of the included studies involved large numbers of patients and healthy controls, and this may strengthen the power of the pooled results. Thirdly, there was no significant publication when tested by Egger and funnel plot, which indicates that our results are stable.

The present study has some limitations. Firstly, the individual studies may have failed to control for potential confounders, which may introduce bias in an unpredictable direction. Secondly, ten of 11 studies were from Asia, and 9 were from China, and thus, more related researches from other countries are wanted to verify the association between geographic location and lung cancer risk.

## Conclusions

Based on the obtained results, we concluded that serum magnesium levels may have no significant association in patients with lung cancer. As we experienced some limitations in our study, such as more studies were from Asia, further studies are wanted to confirm this finding.

## Abbreviations

CI: Confidence intervals
SD: Standard deviation
SMD: Standard mean differences
